# Ab Initio Structure and Dynamics of Beryllium Monofluoride and Its Anion

**DOI:** 10.1021/acs.jpca.4c06334

**Published:** 2024-11-04

**Authors:** Jacek Koput

**Affiliations:** Department of Chemistry, Adam Mickiewicz University, 61-614 Poznań, Poland

## Abstract

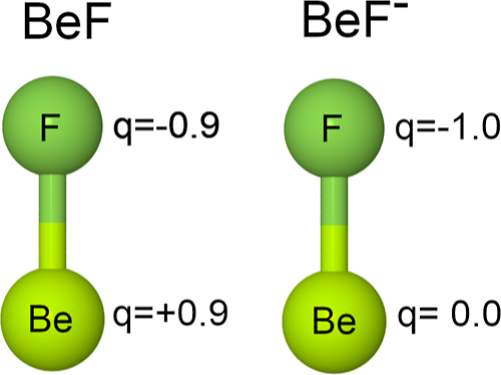

The accurate potential energy functions of beryllium monofluoride, BeF, and its anion, BeF^–^, have been determined from ab initio calculations using the coupled-cluster approach, up to the CCSDTQP level of approximation, in conjunction with the augmented correlation-consistent core–valence basis sets, aug-cc-pCV*n*Z, up to septuple-zeta quality. The vibration–rotation energy levels of the two species were predicted to near the “spectroscopic” accuracy. Changes in the electron density distribution upon formation of the Be–F chemical bond are discussed.

## 1 Introduction

Experimental high-resolution spectroscopic studies on vibration–rotation energy levels of beryllium monofluoride, BeF, are very limited.^1,2^ The vibrational fundamental wavenumber ν of BeF in its X^2^Σ^+^ state was observed to be 1246.70 cm^–1^, and the effective ground-state rotational constant *B*_0_ was determined to be 1.4801 cm^–1^. The spectroscopic constants of its anion, BeF^–^, were reported only recently.^3^ In particular, the electron affinity (EA) of BeF was determined to be 8697(6) cm^–1^ (the number in parentheses being the uncertainty in the last digit quoted). The vibrational fundamental wavenumber of BeF^–^ in the X^1^Σ^+^ state was observed to be 1040(7) cm^–1^.

Theoretical studies, largely on electronic properties of a BeF radical, were more numerous.^4−19^ In the most sophisticated studies to date,^10,11,13^ the multireference configuration interaction method including the Davidson correction (MRCI + Q), in conjunction with the correlation-consistent basis sets up to quintuple-zeta quality, was applied. The calculated molecular descriptors were found to vary substantially depending on the size of the active space and the number of electrons included in the correlation treatment. The equilibrium internuclear distance *r*_e_ and the vibrational fundamental wavenumber ν of a BeF radical in its X^2^Σ^+^ state were predicted to span rather wide ranges 1.348–1.374 Å and 1218–1338 cm^–1^, respectively. The best estimates of the effective rotational constant *B*_0_ were reported in refs (10, 11 and 13) to be 1.4658, 1.466, and 1.450 cm^–1^, respectively. The corresponding best estimates of the vibrational fundamental wavenumber ν were reported to be 1235, 1254, and 1321 cm^–1^, respectively. Concerning the anion BeF^–^, the vibrational harmonic wavenumber ω_e_ was calculated to be 1073.2 cm^–1^ [CCSD(T)/aug-cc-pwCV5Z]^3^ and 1178.2 cm^–1^ [MRCI + Q/aug-cc-pVQZ],^15^ compared to the experimental estimate^3^ of 1059(6) cm^–1^.

The aim of this work is to provide the accurate state-of-the-art potential energy functions for the ground electronic state of BeF and its anion. These functions are calculated using the single-reference coupled-cluster approach, up to the CCSDTQP level of approximation, in conjunction with the augmented correlation-consistent core–valence basis sets up to septuple-zeta quality. By accounting additionally for the scalar relativistic effects, this permitted the prediction the associated vibration–rotation energy levels of both species to near the “spectroscopic” accuracy.

## 2 Results and Discussion

The potential energy functions of BeF and BeF^–^ were determined initially using the single-reference coupled-cluster method accounting for connected single and double excitations and a noniterative correction for connected triple excitations, CCSD(T).^20−26^ For the BeF radical, the spin-restricted coupled-cluster method, RCCSD(T),^24−26^ as implemented in the MOLPRO package of ab initio programs,^27^ is used. In this case, the CCSD wave function was based on spin-restricted open-shell Hartree–Fock (ROHF) molecular orbitals as a reference wave function. Because the MOLPRO package cannot handle functions higher than *i*, the calculations involving such functions were performed with the OpenMolcas package of ab initio programs.^28^ The 1s-like orbital of beryllium was treated as a valence orbital in the correlation treatment. The one-particle basis sets employed were the correlation-consistent basis sets up to septuple-zeta quality, cc-pV*n*Z (*n* = D–7).^29−34^ The basis sets for beryllium were augmented with tight functions (C). The cc-pCV*n*Z basis sets up to quintuple-zeta quality were developed by Prascher et al.^29^ The cc-pCV6Z and cc-pCV7Z basis sets were developed for the previous studies on BeH_2_ and Be_2_.^30,31^ Because both species under consideration were polarized as Be^+^F^–^, the basis sets for fluorine were augmented with diffuse functions (aug). These aug-cc-pV*n*Z basis sets were taken from the literature.^32−34^ The pair of the basis sets: cc-pCV*n*Z for beryllium and aug-cc-pV*n*Z for fluorine, is further referred to as “*n*Z”.

The total energies of BeF and BeF^–^ were calculated at the CCSD(T)/*n*Z (*n* = 5–7) level of theory at 44 internuclear distances ranging from 1.0 to 2.5 Å. The vibration–rotation energy levels were then calculated using the Numerov–Cooley method,^35^ for the rotational quantum number *N* = 0–3. The energy levels were calculated using the nuclear masses of beryllium and fluorine. The predicted molecular parameters are given in Table 1. The parameters quoted include the equilibrium internuclear distance *r*_e_, the total energy at minimum, the binding energy *D*_e_, the vibrational fundamental wavenumber ν, and the effective ground-state rotational constant *B*_0_. For a given vibrational state, the effective rotational constant *B_v_* was determined by fitting the predicted rotation energies with a power series in *N*(*N* + 1). For the X^2^Σ^+^ state of BeF, the molecular parameters predicted with the *n*Z series of basis sets tend to converge. In particular, the electronic total energy of BeF is converged to better than 2 mE_h_. However, this is clearly not the case for the X^1^Σ^+^ state of BeF^–^. Upon enlarging of the *n*Z basis set, incremental changes in the molecular parameters of BeF^–^ were found to be by the order of magnitude larger than the corresponding changes for BeF. Such a sensitivity of the calculated molecular parameters to the basis set was observed earlier for the beryllium monohydride anion, BeH^–^.^36^ To account for the “extra” electron of the BeF^–^ anion, the basis sets should be extended with an another set of diffuse functions.^37^ Therefore, the pair of the basis sets: aug-cc-pCV*n*Z for beryllium and d-aug-cc-pV*n*Z for fluorine, was used. It is further referred to as “a*n*Z”. In fact, as shown in Table 1, incremental changes in the molecular parameters of BeF^–^ along the a*n*Z series of basis sets are similar to the corresponding changes for BeF (along the *n*Z series). For both species under consideration, changes in the predicted equilibrium distance r_e_ and the binding energy *D*_e_ beyond the *n* = 7 basis set are estimated to be about 0.0002 Å and 20 cm^–1^, respectively. The analogous changes in the vibrational fundamental wavenumber ν and the effective rotational constant *B*_0_ are estimated to be about 0.4 and 0.0004 cm^–1^, respectively.

**Table 1:** Molecular Parameters for the X^2^Σ^+^ State of BeF and X^1^Σ^+^ State of BeF^–^ Determined Using the CCSD(T) Method and Various Basis Sets (*n*Z and a*n*Z)

	*n* = 5	*n* = 6	*n* = 7
BeF (*n*Z)			
*r*_e_^a^ (Å)	1.36230	1.36177	1.36160
E + 144^b^ (hartree)	–0.546153	–0.549955	–0.551697
*D*_e_^c^ (cm^–1^)	47,884	47,990	48,016
ν^d^ (cm^–1^)	1246.0	1247.3	1247.8
*B*_0_^e^ (cm^–1^)	1.47747	1.47862	1.47899
BeF^–^ (*n*Z)			
*r*_e_ (Å)	1.41115	1.40971	1.40850
*D*_e_ (cm^–1^)	28,589	28,799	28,934
ν (cm^–1^)	1040.1	1045.0	1048.7
*B*_0_ (cm^–1^)	1.37424	1.37711	1.37951
EA^f^ (cm^–1^)	8159	8347	8479
BeF^–^ (a*n*Z)			
*r*_e_ (Å)	1.40796	1.40738	1.40719
*D*_e_ (cm^–1^)	28,997	29,024	29,036
ν (cm^–1^)	1050.0	1051.1	1051.4
*B*_0_ (cm^–1^)	1.38056	1.38170	1.38208
EA (cm^–1^)	8579	8583	8585

a The equilibrium internuclear distance.

b The total energy at a minimum.

c The binding energy.

d The vibrational fundamental wavenumber.

e The ground-state effective rotational constant.

f The EA.

The potential energy functions obtained with the *n* = 7 basis set were gradually corrected by accounting for the core–electron correlation of fluorine, valence–electron correlation beyond the CCSD(T) level of approximation, and scalar relativistic effects.

The core–electron correlation correction was estimated as a difference in the total energy of BeF/BeF^–^ obtained at the CCSD(T)/5Z level of theory including or not the 1s-like orbital of fluorine in the correlation treatment. The basis set for fluorine was augmented with tight functions.^38^

The effect of valence–electron correlation beyond the CCSD(T) level of approximation was estimated from calculations using the CCSDT, CCSDTQ, and CCSDTQP methods. The higher-order valence–electron correlation correction to the total energy was composed of a sum of three terms: Δ*T* = *E*[CCSDT/QZ] – *E*[CCSD(T)/QZ], Q = *E*[CCSDTQ/TZ] – *E*[CCSDT/TZ], and P = *E*[CCSDTQP/DZ] – *E*[CCSDTQ/DZ], where *E*[···] denotes the total energy of BeF/BeF^–^ at a given level of theory. The first term accounts for a difference between the iterative and perturbational treatment of connected triple excitations. The second and third terms account for contributions due to connected quadruple and pentuple excitations, respectively. The calculations were performed using the MRCC program.^39^

The scalar relativistic correction was estimated using the exact-2-component (X2C) approach.^40^ It was determined as a difference in the total energy of BeF/BeF^–^ calculated using either the X2C or nonrelativistic Hamiltonian, both energies obtained at the CCSD(T)/5Z(uncontracted) level of theory.

**Figure 1:**
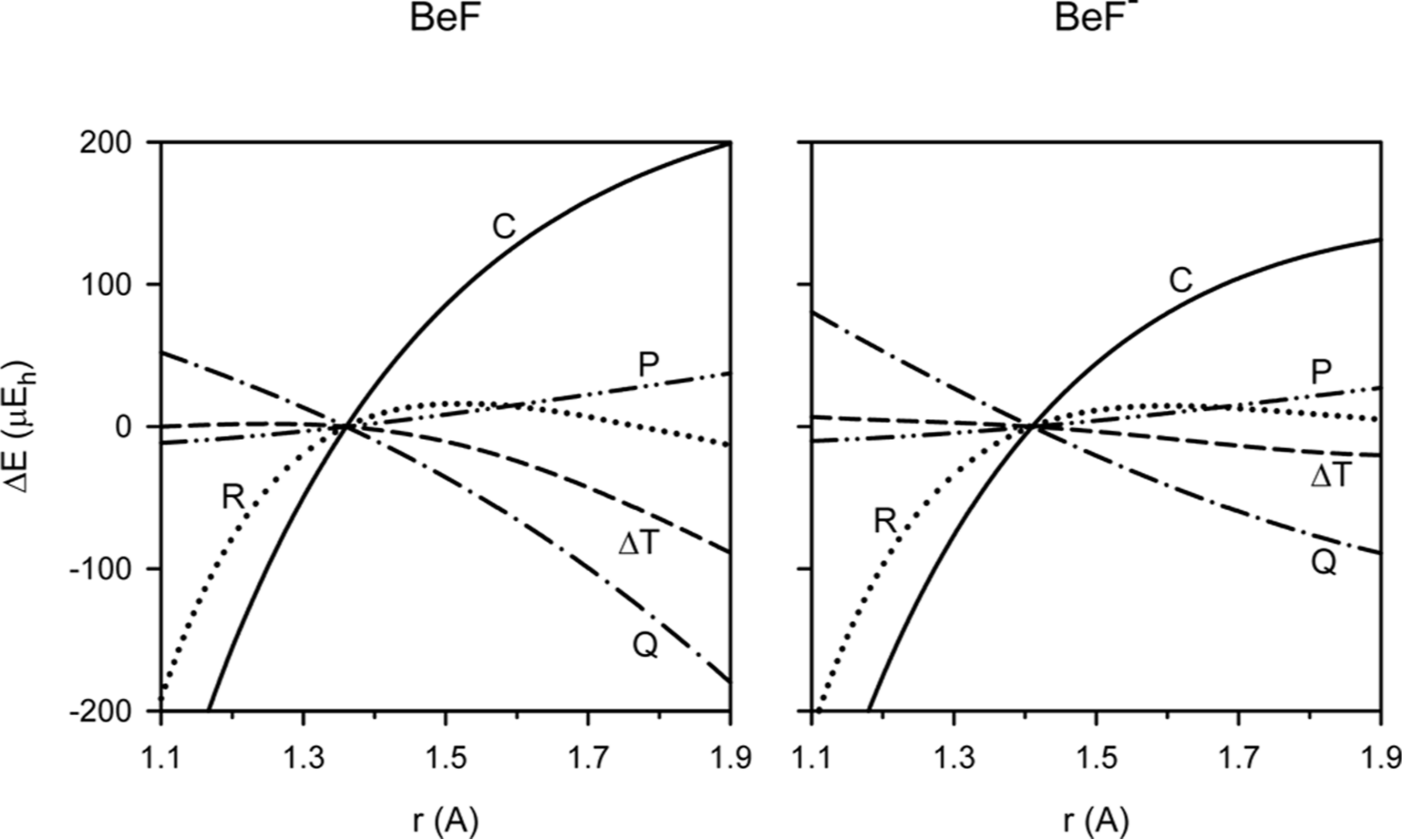
Changes in the total energy Δ*E* for the X^2^Σ^+^ state of BeF and the X^1^Σ^+^ state of BeF^–^ due to fluorine core–electron correlation (C solid line), higher-order valence–electron correlation (Δ*T* dash line, Q dash-dot line, P dash-dot-dot line) and the scalar relativistic effect (R dotted line) as functions of the internuclear distance *r*. The relative values of Δ*E* are plotted; the origin of the Δ*E* axis are the absolute changes Δ*E* calculated at *r* = 1.36 and 1.41 Å for BeF and BeF^–^, respectively.

The corrections to the total energy of BeF and BeF^–^ due to core–electron correlation of fluorine (C), high-order valence–electron correlation (Δ*T*, Q, P), and scalar relativistic (R) effects as functions of the internuclear distance *r* are illustrated in Figure 1. In the vicinity of the equilibrium configuration of BeF/BeF^–^, the corrections C and R amount to about −64.2 and −89.6 mE_h_, respectively, being essentially the same for both species. The corrections Δ*T*, Q, and P amount to about −40/–60, −230/–400, and 20/20 μE_h_, respectively. For BeF/BeF^–^, contributions to the correlation energy due to connected triple excitations were calculated at the CCSDT/QZ level of theory to be about −10,200/–12,100 μE_h_. For both species, the difference between the iterative and perturbational treatment of connected triple excitations is thus unimportant. Note, however, that the dependence of the corrections Δ*T*, Q, and P on the internuclear distance r is stronger for the BeF radical than for the BeF^–^ anion. This is related to the well-known inability of the reference ROHF wave function to describe dissociation of a neutral molecule into open-shell fragments. The BeF radical in its X^2^Σ^+^ state dissociates to give the atoms Be(^1^S) and F(^2^P). In contrast, the BeF^–^ anion in its X^1^Σ^+^ state dissociates to give the atom Be(^1^S) and the anion F^–^(^1^S). The higher-order valence–electron correlation corrections to the total energies of BeF and BeF^–^ are dominated by a contribution due to connected quadruple excitations.

For each of the corrected potential energy functions of BeF and BeF^–^, the molecular parameters were determined, and the results are given in Table 2. All of the above-mentioned corrections to the molecular parameters of BeF and BeF^–^ were found to be small and tend to cancel each other. The best predicted Born–Oppenheimer potential energy function of BeF in its X^2^Σ^+^ state has a minimum at 1.3610(2) Å and the well depth of 48,038(20) cm^–1^. The former value is close to its experimental counterpart, derived by Walker and Barrow^1^ to be 1.3609 Å. The latter value should be further corrected for the spin–orbit effects of the fluorine atom in its ground electronic state ^2^P. Using the experimental hyperfine splittings measured by Laguna and Beattie,^41^ the binding energy D_e_ of BeF is finally estimated to be 47,903(20) cm^–1^. The potential energy function of BeF^–^ in its X^1^Σ^+^ state was predicted to be shallower, with a minimum at 1.4066(2) Å and the well depth of 29,121(20) cm^–1^. The corresponding vibrational fundamental wavenumbers ν for BeF and BeF^–^ were calculated to be 1247.4(4) and 1051.4(4) cm^–1^, respectively. The best predicted potential energy functions for both species are given in Table S1 of the Supporting Information. These functions were used to determine the corresponding vibration–rotation energies, and the calculated vibrational term values *G_v_* and the effective rotational constants *B_v_* of low-lying energy levels of BeF and BeF^–^ are given in Table 3. The vibration–rotational constants predicted for the X^2^Σ^+^ state of BeF can be compared with the experimental data. Walker and Barrow^1^ reported these spectroscopic constants to be *G*_1_ = 1246.4 cm^–1^; *B*_0_ = 1.4801 and *B*_1_ = 1.4625 cm^–1^. The corresponding constants were reported by Tai and Verma^2^ to be *G*_1_ = 1246.70 and *G*_2_ = 2474.55 cm^–1^; *B*_0_ = 1.4801, *B*_1_ = 1.4615, and *B*_2_ = 1.4428 cm^–1^. Using the data of Table 3, the harmonic wavenumber ω_e_ and the anharmonicity constant ω_e_*x*_e_ of BeF are predicted to be 1266.24 and 9.27 cm^–1^, respectively. The corresponding vibrational constants were derived by Tai and Verma^2^ to be 1265.54(10) and 9.422(28) cm^–1^, respectively. Concerning the X^1^Σ^+^ state of BeF^–^, only the value of *G*_1_ = 1040(7) cm^–1^ was reported by Green et al.^3^ From the analysis of the photodetachment spectra of BeF^–^, the vibrational constants were derived to be ω_e_ = 1059(6) cm^–1^ and ω_e_*x*_e_ = 9.5(18) cm^–1^. The corresponding constants were estimated in this work to be 1075.23 and 11.84 cm^–1^, respectively.

**Table 2:** Molecular Parameters^a^ for the X^2^Σ^+^ State of BeF and X^1^Σ^+^ State of BeF^–^ Determined Using Various Potential Energy Functions

	V^b^	V + C^c^	V + C + H^c^	V + C + H + R^c^
BeF (*n*Z)				
*r*_e_ (Å)	1.36160	1.36104	1.36120	1.36102
E + 144 (hartree)	–0.551697	–0.615873	–0.616127	–0.705771
*D*_e_ (cm^–1^)	48,016	48,046	48,040	48,038
ν (cm^–1^)	1247.8	1248.8	1247.8	1247.4
*B*_0_ (cm^–1^)	1.47899	1.48022	1.47984	1.48023
BeF^–^ (a*n*Z)				
*r*_e_ (Å)	1.40719	1.40658	1.40681	1.40661
*D*_e_ (cm^–1^)	29,036	29,063	29,120	29,121
ν (cm^–1^)	1051.4	1052.4	1051.6	1051.4
*B*_0_ (cm^–1^)	1.38208	1.38327	1.38280	1.38321
EA (cm^–1^)	8585	8582	8624	8627

a See Table 1.

b The potential energy functions were calculated at the CCSD(T)/*n* = 7 level of theory.

c Including additional corrections for the fluorine core–electron correlation (C), higher-order valence–electron correlation (H), and scalar relativistic (R) effects (see text).

**Table 3:** Predicted Vibrational Term Values (*G*_v_) and Effective Rotational Constants (*B*_v_, all in cm^–1^) for the X^2^Σ^+^ State of BeF and X^1^Σ^+^ State of BeF^–^

	BeF		BeF^–^	
*v*	*G_v_*	*B_v_*	*G_v_*	*B_v_*
0	0^a^	1.48023	0^a^	1.38321
1	1247.4	1.46243	1051.4	1.36120
2	2476.4	1.44481	2079.1	1.33923
3	3687.2	1.42736	3083.4	1.31730
4	4880.2	1.41009	4064.4	1.29541
5	6055.4	1.39298	5022.3	1.27354

a The zero-point energies for BeF and BeF^–^are 631.1 and 534.8 cm^–1^, respectively.

Using the vibrational zero-point energies calculated for BeF and BeF^–^, the EA of BeF in its X^2^Σ^+^ state is predicted in this work to be 8723 cm^–1^, being slightly larger than its experimental counterpart of 8697(6) cm^–1^ determined by Green et al.^3^ The dissociation energies *D*_0_ of BeF and BeF^–^ are predicted to be 47,272 and 28,586 cm^–1^, respectively. The experimental *D*_0_ value for BeF is rather uncertain,^42^ estimated to be 48,200(800) cm^–1^ [5.98(10) eV]. For BeF^–^, Green et al.^3^ estimated only the lower bound to *D*_0_ to be 28,460 cm^–1^. On the other hand, the difference in the dissociation energies *D*_0_(BeF) – *D*_0_(BeF^–^) was found in that study to be 18,735(6) cm^–1^, compared to the present theoretical value of 18,686 cm^–1^.

**Figure 2:**
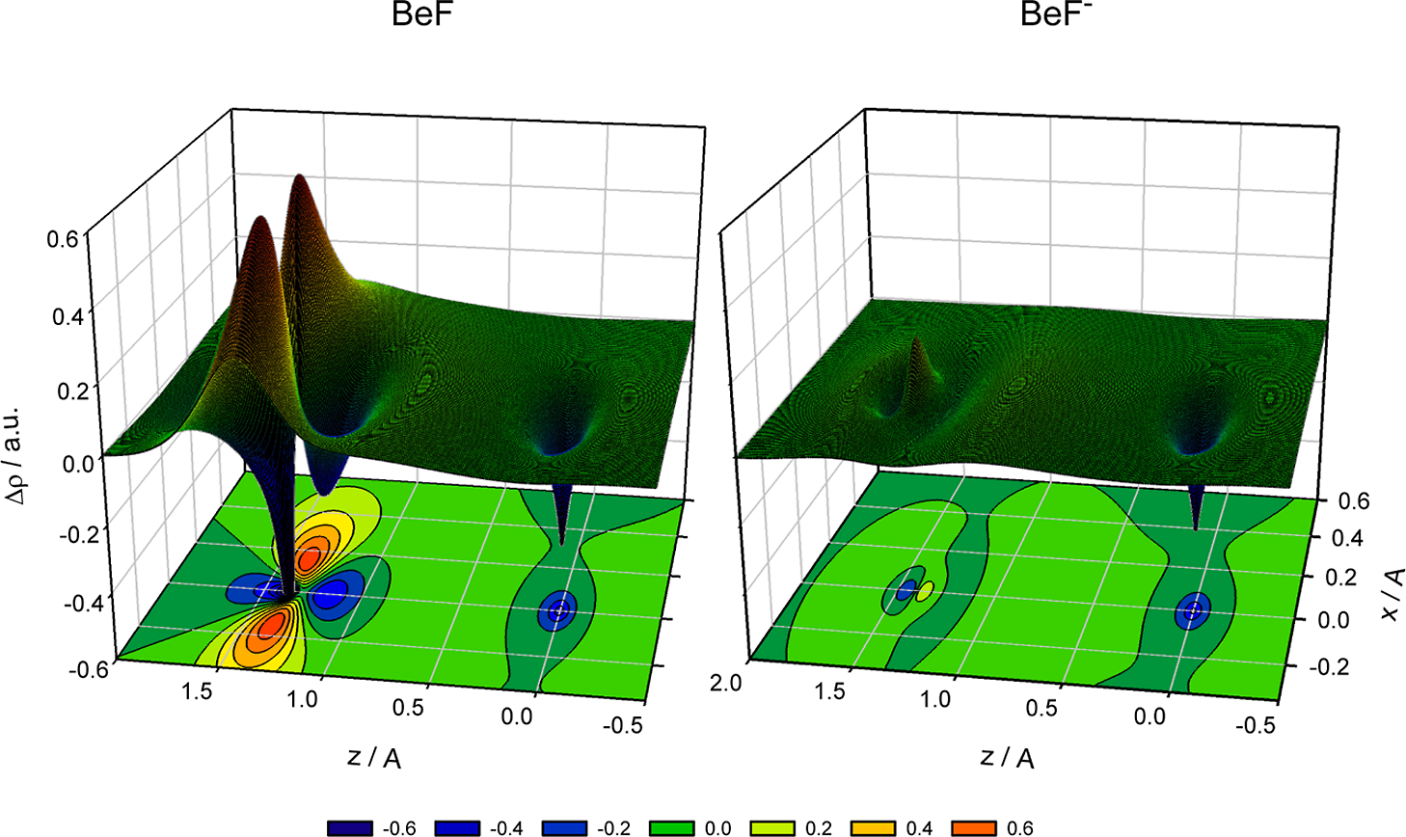
Density difference functions Δρ(*x*, 0, *z*) for the X^2^Σ^+^ state of BeF and the X^1^Σ^+^ state of BeF^–^. The Be and F nuclei are located at the *z* axis (Be at *z* = 0 Å). Regions that locally lose or gain the electron density are shown with blue to red, respectively.

**Figure 3:**
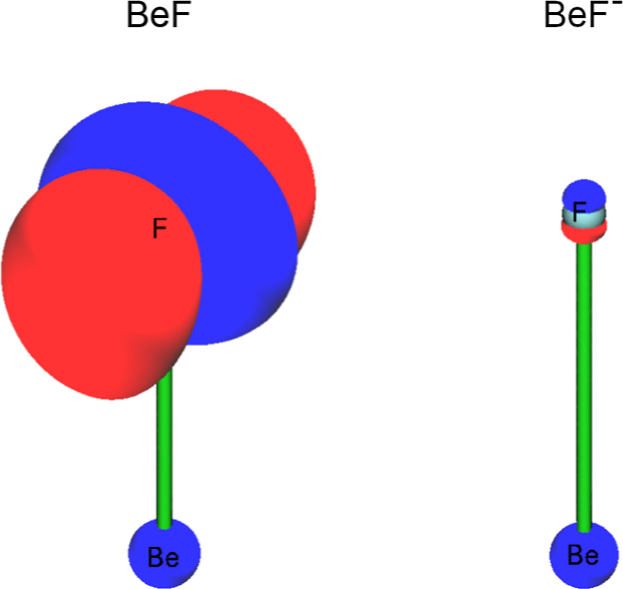
Isosurfaces of the density difference functions Δρ(*x*, *y*, *z*) = *c* for the X^2^Σ^+^ state of BeF and the X^1^Σ^+^ state of BeF^–^. The Be and F nuclei are located at the *z* axis, *c* = 0.05 a.u.

**Figure 4:**
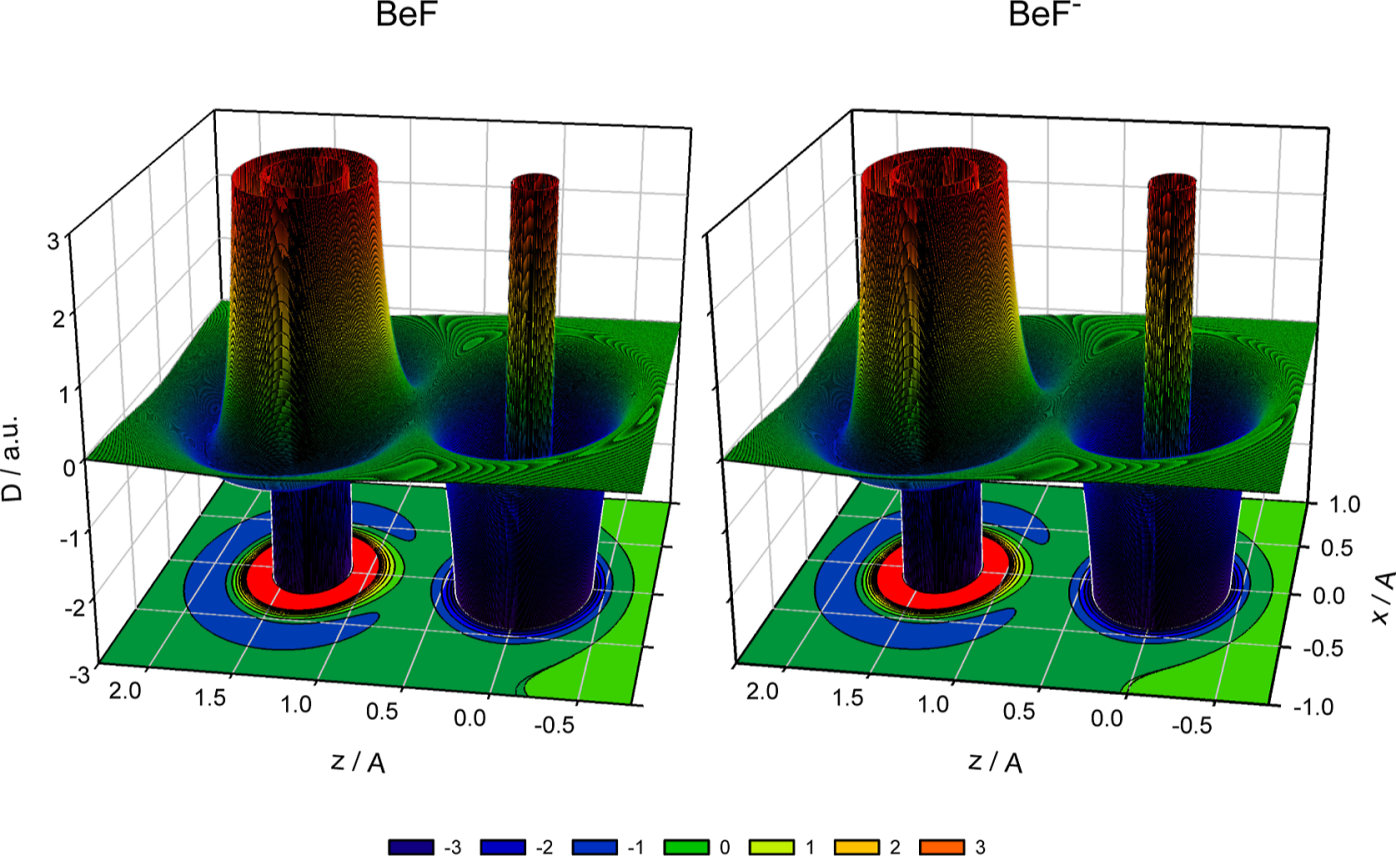
Laplacian functions *D* = −∇^2^ρ(*x*, 0, *z*) for the X^2^Σ^+^ state of BeF and the X^1^Σ^+^ state of BeF^–^. The Be and F nuclei are located at the *z* axis (Be at *z* = 0 Å). Regions of local depletion or concentration of the electron density are shown with blue to red, respectively. Function values beyond ±3 a.u. are not shown; the *D* values span the range [−2 × 10^3^, 2 × 10^6^] a.u.

To gain insight into the nature of a chemical bond in BeF and BeF^–^,^3,14,16−19^ net atomic charges and electron densities were determined using the multireference averaged coupled-pair functional (MR-ACPF) method^43^ with the aQZ basis set. The calculations consisted of three steps: the Hartree–Fock step, the complete-active-space self-consistent-field step, and the MR-ACPF step. The usual full-valence active space, including the 2s- and 2p-like orbitals of the beryllium and fluorine atoms was used. In this way, both static and dynamic electron correlation effects were taken into account. Both canonical (delocalized) and natural bond (localized) orbitals (NBO)^44^ were calculated. The atoms-in-molecules (AIM)^45^ and electron-localization-function (ELF)^46,47^ approaches were also applied, as implemented in the Multiwfn program.^48^ For the BeF radical in its X^2^Σ^+^ state, the net charge on the Be center was determined to be about +0.94*e*, almost independent of the type of the population analysis and on accounting for electron correlation. For the BeF^–^ anion in its X^1^Σ^+^ state, the net charge on the Be center was determined to be only about +0.05*e* using the NBO and AIM approaches, whereas about −0.50*e* using the Mulliken-type population analysis. The predicted NBO and AIM net atomic charges indicate thus that upon formation of the Be–F chemical bond, the electronic charge of almost one *e* is shifted from the Be center to the F center for the BeF radical, whereas there is almost no electron density redistribution for the BeF^–^ anion. Changes in the electron density distribution upon formation of the Be–F bond can be characterized by the density difference function Δρ = ρ(BeF/BeF^–^) – [ρ(Be) + ρ(F/F^–^)], where ρ(BeF/BeF^–^) is the electron density of the BeF or BeF^–^ molecule, ρ(Be) and ρ(F/F^–^) are the electron densities of the respective atomic fragments. The function Δρ illustrates how electrons rearrange upon formation of a chemical bond, and it is equivalent to the density difference function Δρ_SA_ discussed by Bader et al.^49^ Two-dimensional slices of the functions Δρ(*x*, 0, *z*) calculated for BeF and BeF^–^ at the MR-ACPF/aQZ level of theory are shown in Figure 2. Both molecules are located along the *z* axis. As illustrated, in the vicinity of the Be nucleus and along the Be–F bond path, the density difference functions of the BeF and BeF^–^ molecules are similar in appearance to each other. However, in the vicinity of the F nucleus, the density difference functions are in a sharp contrast. This is more evidenced by the three-dimensional isosurface functions Δρ(*x*, *y*, *z*) = *c*, where *c* is a constant, shown in Figure 3. For both BeF and BeF^–^ molecules, there is the region of a similar loss of charge near the Be nucleus. For the BeF radical, there is a large change in the electron density near the F nucleus. It encompasses a depletion of charge in the direction parallel to the Be–F bond path and an accompanying concentration of charge in the perpendicular direction. This is largely a result of change in the electron density of the singly occupied 2p-like orbital of the fluorine atom upon formation of the Be–F bond. For the BeF^–^ anion, the change in the electron density near the F nucleus is barely visible. This is consistent with the NBO and AIM net atomic charges for BeF and BeF^–^ discussed above. The distribution of electronic charge for BeF and BeF^–^ is further characterized in Figure 4, where two-dimensional slices of the Laplacian functions −∇^2^ρ(*x*, 0, *z*) are shown. For the BeF radical, the shape of the Laplacian surface indicates a substantial charge concentration near the F nucleus, accompanied by a corresponding charge depletion near the Be nucleus. This shape is characteristic of the F^–^ anion and the Be^+^ cation,^45^ thus confirming the considerable polarization of charge upon formation of the Be–F bond. Such a large charge transfer to the fluorine center was regarded by Bader^45^ as the major source of chemical bonding in various fluorides. Somewhat surprisingly, the shape of the Laplacian surface for the BeF^–^ anion was found to be very much alike. The only plausible explanation, consistent with the net atomic charges and the shape of the function Δρ(*x*, *y*, *z*) predicted for BeF^–^, is that upon formation of the Be–F bond, the donation of the electronic charge of about one *e* from the Be center to the F center is accompanied by the back-donation of the “extra” electron of the F^–^ anion. To quantify properties of the Be–F bond, various AIM descriptors were determined for the BeF and BeF^–^ molecules. Some of the AIM descriptors for BeF^–^, along with results of the ELF analysis, were recently discussed in detail by Guha.^18,19^ The AIM descriptor values were calculated at the bond critical point (3, −1), situated for both species on the *z* axis, about 0.5 Å from the Be nucleus. These values are given in Table 4. All of the calculated AIM descriptors appeared to be essentially the same for both the BeF and BeF^–^ molecules and typical of the closed-shell interaction, as defined by Bader and Essén.^50^ The kinetic energy densities per electron *G*/ρ are predicted to be significantly greater than unity and the ratios of the kinetic energy density components *G*_⊥_/*G*_∥_ and of the Hessian eigenvalues |λ_1_|/λ_3_ are small. From the AIM theory perspective, the nature of the Be–F chemical bond in the BeF radical and in the BeF^–^ anion is thus the same. The ELF descriptor values calculated for the BeF and BeF^–^ molecules are given in Table 5. The low value of ELF at the Be–F bond critical point is consistent with the high value of the kinetic energy density *G*. For both species, the three-dimensional ELF exhibits two core and two monosynaptic valence basins Ω(X) (X = Be and F), including core and valence electrons of the Be and F centers, respectively, as well as a single disynaptic valence basin V(Be,F). For both species, the basin V(Be,F) is located around the Be–F bond path and populated by nearly two electrons, with the population variance σ^2^ exceeding one-half of the population value. In general, such a disynaptic valence basin is characteristic of a two-center covalent bond of the Lewis model. Note, however, that the large population variance of the basin V(Be,F), together with large covariance values between the basins V(Be,F) and V(F), indicate significant fluctuations of the electron density between these two basins. And, conversely, there is almost no electron exchange between the other ELF basins. The correlation coefficients between the basins V(Be,F) and V(F) were calculated to be −0.75 and −0.72 for the BeF and BeF^–^ molecules, respectively. This confirms a strong relationship between the electron populations of these two basins. The described features resemble the ELF characteristic of lithium fluoride, LiF, a traditional textbook example of the ionic-type chemical bonding. Assuming the basins V(Be,F) and V(F) can be treated as a union, the following picture of chemical bonding emerges from the predicted ELF basin populations. The bonding arises from the interaction between the F^–^ anion and the Be^+^ cation for the BeF radical, whereas between the F^–^ anion and the neutral Be atom for the BeF^–^ anion.

**Table 4:** AIM Descriptors^a^ (in Atomic Units) of the Be–F Bond for the X^2^Σ^+^ State of BeF and the X^1^Σ^+^ State of BeF^–^

	BeF	BeF^–^
ρ^b^	0.15	0.13
∇^2^ρ^c^	1.40	1.16
*G*^d^	0.38	0.31
*G*_⊥_^d^	0.03	0.02
*G*_∥_^d^	0.33	0.27
*V*^e^	–0.42	–0.34
*H*^f^	–0.04	–0.03
λ_1_ = λ_2_^g^	–0.48	–0.40
λ_3_^g^	2.35	1.96
*G*/ρ	2.48	2.37
*G*_⊥_/*G*_∥_	0.08	0.08
|λ_1_|/λ_3_	0.20	0.20

a Calculated using the MR-ACPF/aQZ natural orbitals, at *r* = 1.36 Å for BeF and *r* = 1.41 Å for BeF^–^.

b The electron density.

c The Laplacian of the electron density.

d The kinetic energy density and its perpendicular and parallel components, respectively.

e The potential energy density.

f The total energy density.

g Eigenvalues of the Hessian of the electron density.

**Table 5:** ELF Descriptors^a^ (in Atomic Units) of the Be–F Bond for the X^2^Σ^+^ State of BeF and the X^1^Σ^+^ State of BeF^–^

	BeF	BeF^–^
ELF^b^	0.10	0.09
N̅ C(Be)^c^	2.05	2.04
N̅ V(Be)^c^	1.00	2.00
N̅ C(F)^c^	2.12	2.11
N̅ V(F)^c^	5.95	6.02
N̅ V(Be,F)^d^	1.84	1.78
σ^2^ V(Be)^e^	0.18	0.26
σ^2^ V(F)^e^	1.18	1.20
σ^2^ V(Be,F)^e^	0.99	0.95
cov[V(Be),V(F)]^f^	–0.07	–0.13
cov[V(Be,F),V(Be)]^f^	–0.05	–0.06
cov[V(Be,F),V(F)]^f^	–0.80	–0.77

a See Table 4.

b The ELF value at the bond-critical point.

c The population of the core (C) and monosynaptic valence (V) basins.

d The population of the disynaptic valence basin.

e The population variance.

f The covariance value between the basins.

The nature of a chemical bond for the BeF^–^ anion was recently a matter of debate.^3,14,16−19^ Green et al.^3^ suggested that the primary bonding interaction in BeF^–^ arised from the donation of electronic charge from the F center to the Be center—the Be ← F^–^ dative bonding. Kalemos^14^ investigated the electronic structure of the BeF and BeF^–^ molecules in terms of the valence-bond theory and concluded that for BeF^–^, the bonding arose from the resonance of the covalent Be^–^(^2^P)–F(^2^P) and ionic Be(^1^S/^1^D)–F^–^(^1^S) Lewis structures. Liu et al.^16^ concluded that the BeF^–^ anion was mainly covalently bonded, with three strongly polarized (toward the Be center) bonds. This unusual bonding picture was further supported by Qin et al.^17^ The studies by Guha^18,19^ questioned the multiplicity of the chemical bond in the BeF^–^ anion. Based on results of the ELF and AIM analyses of the BeF^–^ electronic structure, Guha concluded that the Be–F bond in question was the electron-sharing covalent bond, rather than the dative one, and that there was no difference in bonding between the BeF radical and its anion. While the present study contradicts the former conclusion, it is perfectly in line with the latter one.

## 3 Conclusions

The accurate potential energy functions of the BeF radical and its anion in the corresponding ground electronic states were determined in the state-of-the-art ab initio calculations. The vibration–rotation energy levels of the BeF and BeF^–^ molecules were predicted to near the “spectroscopic” accuracy. The nature of the Be–F chemical bond was discussed. Perhaps paradoxically, the Be–F bond in the BeF^–^ anion turned out to be closer to the traditional ionic-bond limit rather than to the traditional covalent-bond limit, binding the nominally neutral Be atom and the singly charged F^–^ anion.

## Supporting Information Available

The predicted Born–Oppenheimer (V + C + H + R) potential energy functions for BeF in its X^2^Σ^+^ state and BeF^–^ in its X^1^Σ^+^ state (Table S1) (PDF)
