# Supplementary material for: Ab Initio Structure and Dynamics of Beryllium Monofluoride and Its Anion
Source: J Phys Chem A. 2024 Nov 4;128(45):9763–70. doi: 10.1021/acs.jpca.4c06334 (PMC11571204; doi:10.1021/acs.jpca.4c06334)
Supplement: Supplementary file 1 — jp4c06334_si_001.pdf [file jp4c06334_si_001.pdf]

Ab Initio Structure and Dynamics of Beryllium Monofluoride and Its Anion

Jacek Koput
Department of Chemistry, Adam Mickiewicz University, 61-614 Poznan, Poland

Table S1: The predicted Born-Oppenheimer (V+C+H+R) potential energy functions for BeF in its X $^2\Sigma^+$ state and BeF^- in its X $^1\Sigma^+$ state

BeF		BeF^-	
r (Å)	Energy (E_h)	r (Å)	Energy (E_h)
1.000	-114.50985667	1.050	-114.60253608
1.025	-114.54655769	1.075	-114.62970342
1.050	-114.57766766	1.100	-114.65266249
1.075	-114.60391278	1.125	-114.67196558
1.100	-114.62592614	1.150	-114.68809381
1.125	-114.64425841	1.175	-114.70146638
1.150	-114.65938967	1.200	-114.71244854
1.175	-114.67173806	1.225	-114.72135912
1.200	-114.68166771	1.250	-114.72847563
1.225	-114.68949730	1.275	-114.73404054
1.250	-114.69550327	1.300	-114.73826518
1.275	-114.69992716	1.320	-114.74080487
1.300	-114.70298081	1.340	-114.74268895
1.320	-114.70456107	1.360	-114.74399233
1.340	-114.70546599	1.380	-114.74478229
1.350	-114.70568909	1.390	-114.74500386
1.360	-114.70577046	1.400	-114.74511922
1.370	-114.70571842	1.410	-114.74513507
1.380	-114.70554138	1.420	-114.74505720
1.400	-114.70483944	1.430	-114.74489193
1.420	-114.70371872	1.440	-114.74464474
1.440	-114.70222785	1.460	-114.74392552
1.460	-114.70041075	1.480	-114.74293854
1.480	-114.69830626	1.500	-114.74171879
1.500	-114.69594990	1.525	-114.73991446
1.525	-114.69269822	1.550	-114.73784963
1.550	-114.68915672	1.575	-114.73557147

1.575	-114.68537379	1.600	-114.73312142
1.600	-114.68139177	1.625	-114.73053489
1.625	-114.67724650	1.650	-114.72784321
1.650	-114.67297052	1.675	-114.72507334
1.675	-114.66859160	1.700	-114.72224856
1.700	-114.66413424	1.750	-114.71651193
1.750	-114.65506754	1.800	-114.71076306
1.800	-114.64590888	1.850	-114.70509636
1.850	-114.63676428	1.900	-114.69957934
1.900	-114.62771353	1.950	-114.69425888
1.950	-114.61881446	2.000	-114.68916542
2.000	-114.61011056	2.100	-114.67972691
2.100	-114.59340486	2.200	-114.67131835
2.200	-114.57775106	2.300	-114.66391038
2.300	-114.56321104	2.400	-114.65743133
2.400	-114.54980662	2.500	-114.65179114
2.500	-114.53753664		
